# Lumbar Epidural Cystic Lesion Associated With *Finegoldia magna* Discitis Presenting as Cauda Equina Syndrome: A Case Report

**DOI:** 10.1155/crdi/9659608

**Published:** 2026-08-31

**Authors:** Michael Doarn, Jessica Chastain, Tantien Nguyen, Odion Binitie, John Small

**Affiliations:** ^1^ Florida Orthopaedic Institute, Tampa, Florida, USA, floridaortho.com; ^2^ Department of Translational Research, Foundation for Orthopaedic Research and Education, Tampa, Florida, USA, foreonline.org; ^3^ Sarcoma Department, Moffitt Cancer Center, Tampa, Florida, USA, moffitt.org

**Keywords:** cauda equina syndrome, discal cyst, discitis, epidural cyst, *Finegoldia magna*, lumbar spine, spinal infection

## Abstract

**Study Design:**

A case report of a lumbar epidural cystic lesion associated with discitis presenting with cauda equina syndrome.

**Objective:**

To describe a rare presentation of a lumbar epidural cystic lesion associated with *Finegoldia magna* discitis presenting with cauda equina syndrome, discuss diagnostic considerations, and highlight the overlap between cystic lesions and infectious epidural pathology.

**Summary of Background Data:**

Discal cysts are uncommon epidural lesions that typically communicate with the intervertebral disc and present with radiculopathy. Severe neurological deficits are unusual, but infectious causes should remain in the differential when findings are atypical. When cauda equina syndrome is suspected, emergent diagnosis and treatment are imperative.

**Methods:**

A 41‐year‐old male presented with persistent low back pain, bilateral lower extremity radiculopathy, saddle anesthesia, and bowel and bladder changes consistent with cauda equina syndrome. He reported no history of trauma or infectious symptoms. Magnetic resonance imaging revealed a 1.6 × 1.1 cm cystic mass at the L4–L5 disc level that extended into the spinal canal, along with an L5–S1 central disc herniation. He underwent emergent decompression and debridement of the L4–L5 disc space.

**Results:**

Histologic examination showed benign fibrocartilaginous tissue compatible with intervertebral disc material, and cultures grew *Finegoldia magna* (formerly known as *Peptostreptococcus magnus*). His neurologic symptoms and pain improved immediately after surgery, and he was discharged home on postoperative day four. He was followed clinically and with repeat magnetic resonance imaging, which demonstrated resolution of the cyst and discitis.

**Conclusions:**

Lumbar disc–associated epidural cystic lesions are rare but should be considered in the differential diagnoses in patients with lumbar radiculopathy and/or cauda equina syndrome, and urgent decompression should be performed. This case highlights diagnostic overlap between discal cyst and infectious epidural pathology.

**Level of Evidence:**

Level IV.

**Key Points**
• Intervertebral disc cysts occur most commonly in the lumbar spine and usually have neurological sequelae.
• Cauda equina syndrome can result from an intervertebral disc cyst, which should therefore be included in the differential diagnosis.
• When cauda equina syndrome is suspected, magnetic resonance imaging and surgical decompression should be performed emergently.

## 1. Introduction

Discal cysts are uncommon epidural lesions defined by a cystic cavity that communicates with the adjacent intervertebral disc. They are reported almost exclusively in the lumbar spine, most often at the L4–L5 level, and typically present with radiculopathy from nerve‐root compression; severe neurological compromise is unusual [1–9]. The differential diagnosis for a cystic epidural mass is broad and includes synovial cyst, sequestered disc fragment, epidural abscess, and other epidural cystic lesions, several of which can be difficult to distinguish on imaging alone. Because these entities differ substantially in management, correlation of the clinical, radiographic, and operative findings is essential [10–12].

Cauda equina syndrome (CES) is a surgical emergency resulting from compression of the lumbar nerve roots caudal to the conus medullaris. It may present with low back pain; bowel, bladder, and sexual dysfunction; perineal and lower‐extremity paresthesia or anesthesia; and lower extremity weakness [13]. Lumbar disc herniation is the most common etiology, but uncommon lesions can mimic this presentation, which makes clinicoradiologic correlation essential [13–17].

We present a rare case of a ventral epidural cystic lesion associated with *Finegoldia magna* culture‐positive discitis in a patient who presented with CES, and we discuss the diagnostic overlap between discal cysts and infectious epidural pathology.

## 2. Case Presentation

A healthy 41‐year‐old man presented to the clinic with worsening low back pain; numbness in the buttocks and scrotum; and pain, weakness, numbness, and tingling that radiated down both legs and had progressed over the preceding week. He also reported an inability to urinate or move his bowels. He was nonambulatory on presentation and denied fevers, chills, or other constitutional symptoms of infection. There was no reported history of trauma, intravenous drug use, or recent infection. Retrospective inflammatory markers, including C‐reactive protein, erythrocyte sedimentation rate, and white blood cell count, were not available for review. On examination, he had tenderness to palpation over the lumbar spine, decreased rectal tone, bilaterally positive straight‐leg‐raise testing, and decreased sensation in the S1 dermatome and perineum.

**FIGURE 1:**
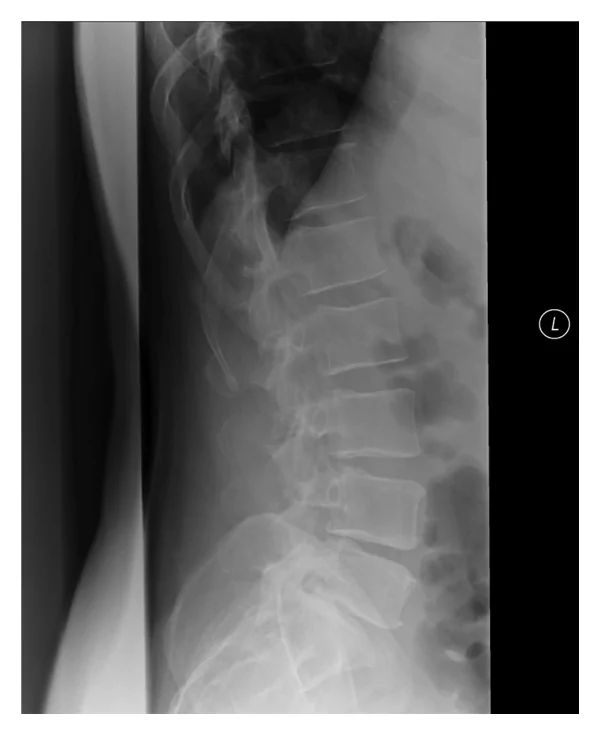
Lumbar spine radiographs demonstrate disc space narrowing at L4–L5 and L5–S1, with no evidence of fracture, spondylolysis, or spondylolisthesis.

**FIGURE 2:**
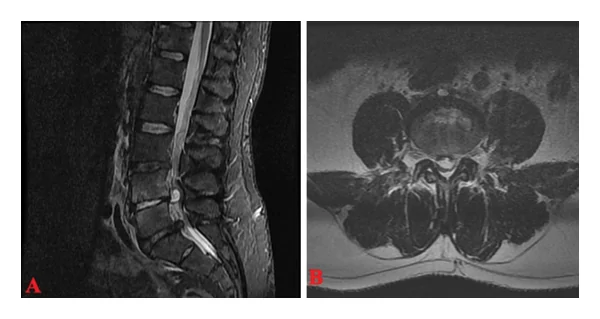
Preoperative magnetic resonance imaging (MRI) revealed a 1.6 × 1.1 cm lesion at the L4–L5 level with associated Type I modic changes (A). Additionally, MRI showed another view of the lesion along with a central disc herniation at L5–S1 causing moderate spinal stenosis (B).

Lumbar radiographs showed disc‐space narrowing at L4–L5 and L5–S1, without fracture, spondylolysis, or spondylolisthesis (Figure 1). The patient was admitted urgently, and magnetic resonance imaging (MRI) demonstrated a large intraspinal, extradural cystic mass with peripheral (rim) contrast enhancement measuring 1.6 × 1.1 cm at the L4–L5 level and causing significant compression of the thecal sac (Figure 2). A central L5–S1 disc herniation producing moderate spinal stenosis was also present. Given the acute CES, he was taken emergently to the operating room for decompression of the L4–L5 and L5–S1 levels.

**FIGURE 3:**
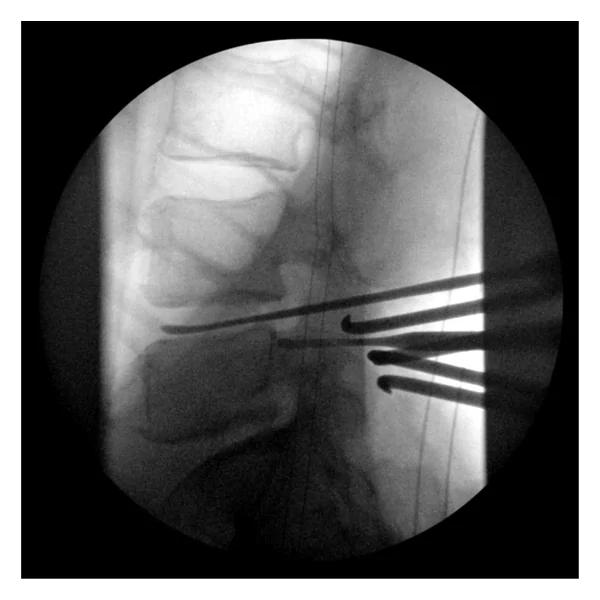
Intraoperative lumbar radiographs demonstrate debridement of the disc space between L4 and L5.

Intraoperatively, a discrete cyst was not visualized; however, a cyst wall communicating with the L4–L5 disc space was identified ventral to the dura. With the patient prone on a Wilson frame, an intraoperative lumbar radiograph showed that the previously collapsed L4–L5 disc space had opened (Figure 3). The L4–L5 disc space was debrided, and cultures together with a 2.5 × 2.0 × 0.3 cm biopsy of the cyst wall and disc space were sent for microbiology and pathology. The L5–S1 disc herniation was also decompressed.

Neurological function and symptoms improved immediately after surgery. Intraoperative cultures grew *Finegoldia magna*. Gross examination of the specimens showed pink‐gray, soft, rubbery tissue, and microscopy revealed benign fibrocartilaginous tissue compatible with intervertebral disc material. A peripherally inserted central catheter was placed, and the infectious disease service directed intravenous antibiotic therapy with ertapenem and vancomycin for treatment of the discitis. The patient was discharged home on postoperative day four. The clinical course is summarized in Table 1.

**TABLE 1:** Clinical timeline from presentation to follow‐up.

Time point	Clinical event and intervention
Presentation (Day 0)	Worsening low back pain, bilateral lower‐extremity radiculopathy, saddle anesthesia, and urinary and bowel retention; nonambulatory. Examination: decreased rectal tone, bilaterally positive straight leg raises, and S1/perineal sensory loss. Lumbar radiographs: disc‐space narrowing at L4–L5 and L5–S1.
Admission (same day)	Urgent hospital admission. MRI: 1.6 × 1.1 cm rim‐enhancing ventral epidural cystic lesion at L4–L5 with thecal sac compression; central L5–S1 disc herniation with moderate stenosis.
Day of surgery	Emergent decompression of L4–L5 and L5–S1. Cyst wall communicating with the L4–L5 disc identified ventral to the dura; disc space debrided; cultures and biopsy obtained. Immediate neurological improvement.
Early postoperative	Cultures grew *Finegoldia magna*; histology showed fibrocartilaginous tissue compatible with disc material. PICC placed; infectious disease–directed intravenous ertapenem and vancomycin for discitis.
Postoperative day 4	Discharged home.
Two‐year follow‐up	MRI showed resolution of the cyst with endplate changes consistent with treated discitis; symptom relief sustained.

Abbreviations: MRI, magnetic resonance imaging; PICC, peripherally inserted central catheter.

**FIGURE 4:**
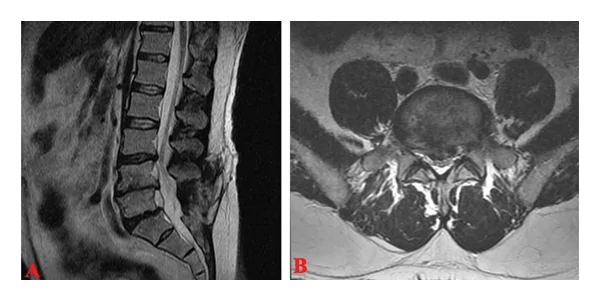
Two‐year postoperative magnetic resonance imaging (MRI) demonstrates resolution of the cyst with vertebral body changes consistent with discitis (A). The L5–S1 intervertebral space shows normal recovery (B).

At 2‐year follow‐up, repeat MRI showed complete resolution of the cyst, with vertebral endplate changes consistent with treated discitis (Figure 4).

## 3. Discussion

The pathogenesis of intervertebral disc cysts remains incompletely understood [1, 4, 5, 18–20]. In this case, the temporal and anatomic association between culture‐positive discitis and the adjacent cystic lesion raises the possibility that the infection contributed to cyst formation; however, the available data do not establish a causal relationship, and this association should be interpreted with caution. *Finegoldia magna*, an anaerobic Gram‐positive coccus, is a recognized cause of mono‐ and polymicrobial soft‐tissue and orthopedic infections [21–23], although its role in spinal infection is infrequently reported [24].

The diagnosis of a discal cyst rests on correlation of the imaging, operative, and histopathologic findings, and this case illustrates the difficulty of meeting that standard with certainty. A defining feature of a discal cyst is demonstrable communication with the intervertebral disc. Although a cyst wall communicating with the L4–L5 disc space was identified intraoperatively, a discrete cyst was not visualized, and histopathology showed only fibrocartilaginous tissue compatible with disc material. These findings are consistent with, but not specific for, a discal cyst, and an organized infectious epidural collection cannot be entirely excluded given the positive *Finegoldia magna* culture. We therefore present this case as an example of the diagnostic overlap between discal cysts and infectious epidural pathology rather than a histologically definitive discal cyst.

Regardless of the ultimate pathologic label, the management principle is unchanged: In suspected CES, emergent MRI followed by decompression and removal of the offending lesion remains the standard of care [13, 17]. This case also highlights the value of a complete infectious workup, including inflammatory markers and culture data; the microbiologic results directed the postoperative antibiotic course and long‐term management.

We suspect that prone positioning on a Wilson frame reopened the collapsed L4–L5 disc space and contributed to dynamic decompression of the cystic collection. The chronic‐appearing, moderate L5–S1 disc herniation was not thought to have contributed to the CES.

This report is limited by its retrospective nature and by the unavailability of preoperative inflammatory markers and complete microbiologic sensitivities, which reflects the age of the case. These limitations should be weighed when interpreting the findings.

## 4. Conclusion

Lumbar disc–associated epidural cystic lesions, although rare, should remain in the differential diagnosis when evaluating a patient with lumbar radiculopathy or CES. Definitive diagnosis requires integration of imaging, pathology, microbiology, and operative findings. This case suggests that discitis should be considered in the evaluation of such discal lesions, and *Finegoldia magna* can be associated with the development of spinal infection.

## Author Contributions

All authors contributed to manuscript preparation, revision, and final approval.

## Funding

No external funding was received.

## Disclosure

CARE Reporting Guideline: This manuscript was revised to align with CARE case report recommendations.

## Ethics Statement

Written informed consent for publication and use of deidentified information and images was obtained from the patient.

## Conflicts of Interest

The authors declare no conflicts of interest.

## Data Availability

Data sharing is not applicable to this article as no datasets were generated or analyzed during the current study.
